# Placenta‐on‐a‐Chip: In Vitro Study of Caffeine Transport across Placental Barrier Using Liquid Chromatography Mass Spectrometry

**DOI:** 10.1002/gch2.201800112

**Published:** 2019-02-18

**Authors:** Rajeendra L. Pemathilaka, Jeremy D. Caplin, Saurabh S. Aykar, Reza Montazami, Nicole N. Hashemi

**Affiliations:** ^1^ Department of Mechanical Engineering Iowa State University Ames IA 50011 USA; ^2^ Petit Institute for Bioengineering and Bioscience Georgia Institute of Technology Atlanta GA 30332 USA; ^3^ Department of Biomedical Sciences Iowa State University Ames IA 50011 USA

**Keywords:** caffeine transport, in vitro, microfluidics, placenta‐on‐a‐chip

## Abstract

Due to the particular structure and functionality of the placenta, most current human placenta drug testing methods are limited to animal models, conventional cell testing, and cohort/controlled testing. Previous studies have produced inconsistent results due to physiological differences between humans and animals and limited availability of human and/or animal models for controlled testing. To overcome these challenges, a placenta‐on‐a‐chip system is developed for studying the exchange of substances to and from the placenta. Caffeine transport across the placental barrier is studied because caffeine is a xenobiotic widely consumed on a daily basis. Since a fetus does not carry the enzymes that inactivate caffeine, when it crosses a placental barrier, high caffeine intake may harm the fetus, so it is important to quantify the rate of caffeine transport across the placenta. In this study, a caffeine concentration of 0.25 mg mL^−1^ is introduced into the maternal channel, and the resulting changes are observed over a span of 7.5 h. A steady caffeine concentration of 0.1513 mg mL^−1^ is reached on the maternal side after 6.5 h, and a 0.0033 mg mL^−1^ concentration on the fetal side is achieved after 5 h.

## 1 Introduction

Caffeine is one of the most popular and widely consumed stimulants across the globe.^1^ Coffee, tea, and cocoa are the primary natural sources of caffeine. Both health authorities and regulatory bodies have raised concerns about consumption of caffeine‐enhanced food and beverages because of the increased availability of caffeine‐enhanced food products containing synthetic caffeine.^2^ Because caffeine is found not only in food and beverages, but also in prescription and over‐the‐counter medications, many pregnant women are very likely to consume caffeine in some form, so they may risk exposing underdeveloped fetuses to this behaviorally active substance. There are concerns that overly heavy caffeine consumption may harm the fetus, since pregnant women require a half‐life 1.5 to 3.5 times greater to metabolize caffeine than non‐pregnant women, possibly causing caffeine to remain in body tissues for longer periods of time. Caffeine has been found on locations within the fetal compartment, suggesting caffeine transport across the placental barrier, and since a fetus would have difficulty in metabolizing caffeine due to lower levels of enzyme production in its developing liver, there is a real possibility that caffeine exposure could damage the fetus.^1^, ^3^ Because of undetermined effects from maternal caffeine intake on the fetus and the increased number of caffeine products available for prenatal consumption, health authorities such as the World Health Organization (WHO) and the Food and Drug Administration (FDA) have developed recommendations restricting caffeine intake during pregnancy and declaring caffeine consumption during pregnancy a global healthcare problem.^3^, ^4^, ^5^

Over the last decade, organ‐on‐a‐chip technology has grown to become one of the most popular alternatives for drug testing and toxicology in vitro. Its aim is to create a 3D microenvironment reminiscent of specific human organs as a means for replicating their functionality.^6^ The placenta has been one of the most difficult organs to replicate using organ‐on‐a‐chip because it is a temporary organ that develops only during pregnancy and changes its structure and functionality over the course of the gestational period. To study this vital organ, in vivo, ex vivo, and in vitro tests have been conducted. In vivo studies conducted on rats and baboons have yielded inconsistent results.^7^, ^8^, ^9^ It is difficult to transfer the results from an animal testing model to a human model because placenta development is different in different mammals.^10^, ^11^, ^12^, ^13^ For example, in humans, glucose transport through the placental barrier is mediated by GLUT1 glucose transporters, while in mice it is mediated by GLUT3 transporters.^14^ This difference shows why experiments using mice can yield lower glucose transfer rates than those found in similar experiments using human placentas. Previous studies have reported that in vitro microphysiological placenta systems are capable of exhibiting similar levels of glucose transport as those of actual placenta.^14^, ^15^ Moreover, in another study, the transport of two xenobiotic substances, heparin and glyburide, was studied using a bioengineered placental model, demonstrating the capability of a placenta‐on‐a‐chip model to successfully mimic at least some of the physiological functions of the human placenta.^16^ It has also been shown that these in vitro models are capable of mimicking placental inflammatory behavior from a placenta attacked with a bacterial infection.^17^

Even though humans are closely related to many other mammals, there is no yolk sac placentation in humans, and an allantoic stalk rather than an allantoic sac is present in a human placenta.^18^ This makes it challenging to compare the results of experiments conducted on placentas of other mammals to results of studies conducted on human placentas. Ex vivo studies in humans have also been conducted by obtaining placentas immediately after childbirth or cesarean sections.^19^ These human studies only provide opportunities to study placentas and gain insight from women in their final term of pregnancy. Difficulty in obtaining consent from women for such participation in such studies and gaining access to placentas before they become no longer viable makes these studies difficult to conduct. Moreover, ethical issues may limit experimental research of this type, and observational studies may be difficult to perform because the women could be from a group self‐selected for testing a particular drug.^20^

During pregnancy, both endogenous substances and xenobiotic substances consumed by a pregnant woman can pass through the placental barrier, possibly causing severe damage to a fetus either before or after birth. For example, an exogenous compound with the chemical nomenclature 1,3,7‐Trimethylpurine‐2,6‐dione, commonly known as caffeine, is quite often consumed worldwide by pregnant women on a daily basis by way of ingesting coffee, tea, energy drinks, chocolate, etc., because it acts as a stimulant for the central nervous system (CNS).^20^ Unfortunately, it has been found that such increased intake of caffeine by pregnant women can result in birth weight (BW) reduction in newborn children, or in reduced neonate size for its gestational age (SGA).^20^, ^21^, ^22^ A meta‐analysis of 32 studies suggests that caffeine intake is associated with an increased risk for reduction in BW, and another meta‐analysis of 26 studies appeared to show a 43 g weight reduction in newborn children whose mothers appeared to be heavy caffeine consumers.^21^, ^22^ Despite these findings, it has never been recorded that caffeine directly causes BW reduction, also known as intrauterine growth retardation (IUGR).^23^, ^24^, ^25^, ^26^, ^27^, ^28^ While both meta‐analyses exhibited some correlation with caffeine intake and BW, inconsistencies found in these studies suggest that there remains a need to identify a proper method for measuring exact caffeine levels crossing the placental barrier when a prospective mother consumes a certain amount of caffeine. Note that the effects on a fetus due to excessive intake of caffeine by pregnant women are beyond the scope of this study because it primarily focuses on the amount of caffeine infiltrating a placental barrier.

Caffeine is easily absorbed by the placental barrier and crosses the barrier freely.^23^ Since the primary enzyme responsible for caffeine metabolization, cytochrome P450 1A2, is absent both from the placenta and the fetus, the rate of metabolism of caffeine depends totally on the metabolization capacity of pregnant women.^24^ One study states that the half‐life of caffeine has a range of 6–16 h in pregnant women compared to that for non‐pregnant women, i.e., 2–8 h.^25^, ^26^ According to guidelines provided by the WHO and FDA, the intake of caffeine during pregnancy should not exceed 300 mg per day, making it necessary to measure a pregnant woman's concentration of caffeine perfused into the fetus in relation to her total caffeine intake.^4^, ^29^

Our placenta‐on‐a‐chip device is designed to represent the trophoblastic epithelium and endothelium of the maternal interface and the fetal interface in a human placenta, respectively. The chip was designed to carry two cell lines to represent both the maternal and the fetal sides. A porous membrane was placed between the two channels to serve as a barrier between the two bloodstreams. This membrane acts as an extracellular matrix (ECM) to provide support for surrounding cells used in our design. Human umbilical vein endothelial cells (HUVECs) and trophoblasts cells (BeWo) were respectively chosen to represent the endothelium in the fetal interface and the epithelium in the maternal interface. This work will enable us to establish a platform for studying the pharmacokinetics of different xenobiotic drugs across the placental barrier, and also enable us to examine the safety of drugs administered to pregnant women.

## 2 Results and Discussion

### 2.1 Cell Growth and Characterization on Membrane

**Figure 1:**
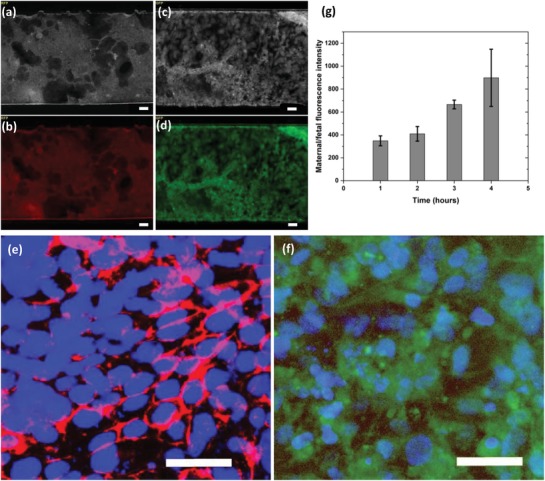
Cells in channels after 48 h of media perfusion. a) BeWo cells with RFP grayscale imaging, b) BeWo cells with RFP color imaging, c) HUVECs with GFP grayscale imaging, d) HUVECs with GFP color imaging, e) BeWo cells showing epithelial adherence junctions with E‐Cadherin and Nuclei labeled with DAPI staining, f) HUVECs showing endothelial adherence junctions with VE‐Cadherin and Nuclei labeled with DAPI staining, scale 50 microns and g) fluorescence intensity measured using dextran for 4 h, data represented as a fraction of maternal intensity/fetal intensity. *n* = 3 independent experiments. Data are presented as mean (±SD).

The HUVECs and BeWo cocultured microfluidic device provided a relevant environment for representing the propagation of a human placenta. The human placenta in vivo consists of three main parts: the epithelium, the endothelium, and the placental barrier. As intended, we were able to replicate an in vivo–like microsystem with HUVECs representing the endothelium, BeWo cells representing the epithelium, and a semipermeable membrane representing the placental barrier. CellTracker results (**Figure** **1**a–d) showed a proliferation of cells over time and cell characterization was used to further study the formation of a placental barrier–like interface used to replicate and mimic placenta‐related physiology. During medium perfusion, cells were able to cover the entire area of both sides of the membrane within 24–30 h from the cell seeding. Cell adhesion on the porous membrane is an important step in properly representing each cell line, and ECM macromolecules play an important role in proper growth and normal function of primary cells.^30^ The most important cell‐adhesion control variable was the cell adhesion time. Various time periods were tested to identify the optimal time for cells to reach solid attachment. Another important parameter affecting cell viability on the channel was the flow rate. Since high flow rates produce high levels of shear stress on the channel walls, and can thereby force attached cells to detach from the membrane and flush out of the device, we tested different flow rates to seek the best results while also satisfying the previously discussed conditions. In the upper channel where BeWo cells were introduced, cells began forming a 3D structure and thereby affected long‐term cell growth in the upper channel. As cell coverage increased, the space remaining for the medium to cross the channel had decreased, causing medium flow to exert pressure on the cells.

When fabricating a placental‐barrier‐like semipermeable membrane, it is important to verify the formation of tight cell–cell junctions. E‐cadherin is considered to be an important molecule when seeking to maintain cell–cell adhesion in the epithelial cell layer because it is restricted to regions of adherence junctions.^31^ We used E‐cadherin present on trophoblast cells to validate the formation of tight junctions and strong cell–cell adhesion in the epithelium. After 3 days, BeWo cells were stained with anti‐E‐cadherin and scrutinized for red fluorescent protein (RFP). As shown in Figure 1e, BeWo cell–cell boundaries tested positive when stained for E‐cadherin, verifying existence of tight junctions across the epithelial cell layer. Tight junctions in the endothelial cell layer ensure tissue integrity and play a vital role in maintenance and control of endothelial cell contacts.^32^ VE‐cadherin was used to investigate cell–cell interactions and the formation of tight junctions on HUVECs that represent the endothelium. Similarly, after 3 days of medium perfusion, HUVECs were marked with anti‐VE‐cadherin and analyzed for green fluorescent protein (GFP). As shown in Figure 1f, VE‐cadherin was detected on cell–cell partitions, verifying the occurrence of tight junctions in the endothelial cell layer. The E‐cadherin and VE‐cadherin‐labeled cell–cell boundaries implied the formation of tight junctions and verified that both the epithelial and endothelial cell layers consisted of a confluent monolayer of cells on the membrane.

Placental barrier permeability was evaluated using 3000 MW fluorescein–dextran anionic probes. When dextran was introduced to the maternal side, fluorescence intensities on both the maternal side and the fetal side were recorded, and the data represented as a fraction, with maternal intensity the numerator and fetal intensity the denominator, as shown in Figure 1g. We observed that, while maternal fluorescence increased over time due to the dilution of the dextran‐mixed medium by the remaining medium in the channels and by the tubing, fetal fluorescence intensity remained at a lower level. Even though a few molecules were diffused from the maternal side to the fetal side across the membrane, overall fetal intensity remained insignificant over time, verifying the integrity of the placental‐barrier‐like semipermeable membrane.

### 2.2 Quantitative Analysis of Caffeine Transport

#### 2.2.1 Concentration of Caffeine Transported through Placental Barrier

**Figure 2:**
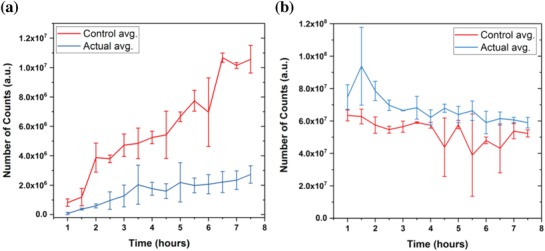
Area under the curve for each chromatogram from LCMS, which were generated for each sample collected from both the maternal and fetal outlet after every 30 min. a) Chromatogram area output for EGM (fetal side). b) Chromatogram area output for F‐12K (maternal side). Actual tests have both cells in the chip and the control has just the bare membrane with media perfusing through the channels. *n* = 3 independent experiments. Data are presented as mean (±SD).

Before calculating caffeine concentrations, we plotted the data obtained from the area under the curve for each chromatogram with respect to time, as shown in **Figure** **2**, and the fetal side (Figure 2a) of the control (samples collected from a chip consisting of a bare membrane with perfusing EGM and F‐12K) showed more fluctuation in terms of the number of counts (representing the area) with a positive gradient with respect to time up to *t* = 6.5 h. Between *t* = 6.5 h and *t* = 7.5 h, concentrations (represented by the number of counts) sought to reach a steady‐state while achieving a peak‐level of caffeine diffusion through the placental barrier. Conversely, the actual data (from chips with cells and medium) show less data variability, with a positive gradient, but data remained in a lower range than in the controlled tests. The actual data also exhibited reaching a peak diffusion between *t* = 5 h and *t* = 7.5 h. On the maternal side (Figure 2b), control data always remained lower than actual data, but it exhibited greater fluctuation than the actual data while the system was moving toward its optimum diffusion stage, and this trend was also observed on the fetal side. The data show attainment of steady‐state between 6 and 7.5 h for actual data.

Using data obtained from both the maternal and fetal calibration curves, individual quadratic curves were fitted and equations with a 95% confidence level found for them. Equations (1) and (2) represent curves fitted for EGM (fetal) and F‐12K (maternal), respectively.

(1) $$A_{f}\;=\;-8.06e^{8}C_{f}^{2}\;+\;5.80e^{8}C_{f}\;+\;3.46e^{5}$$

(2) $$A_{m}\;=\;-2.04e^{8}C_{m}^{2}\;+\;3.97e^{8}C_{m}\;+\;4.64e^{6\;}$$

where *A* and *C* represent the area under the curve from the liquid chromatography mass spectrometry (LCMS) method and the calculated caffeine concentration, respectively. Roots were obtained from each equation for both maternal and fetal caffeine transport. We neglected one root under the condition: *C* ≤ 0.25 mg mL^−1^ because the highest caffeine concentration introduced was 0.25 mg mL^−1^ on the maternal side.

**Figure 3:**
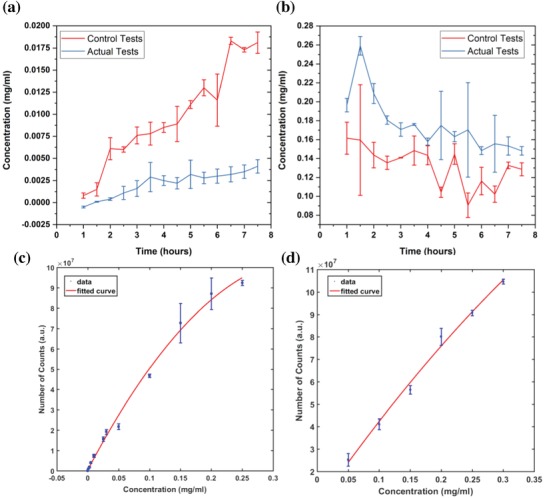
Caffeine concentrations calculated for both the maternal and fetal sides. a) EGM (fetal side). b) F‐12K (maternal side). Actual tests have both HUVECs and BeWo cells on the chip and the control has solely the bare membrane with media perfusing through the channels. c) Calibration curve for caffeine concentrations in EGM (concentrations ranging from 0.00001 to 0.25 mg mL^−1^). d) Calibration curve for caffeine concentrations in F‐12K (concentrations ranging from 0.05 to 0.3 mg mL^−1^). *n* = 3 independent experiments. Data were presented as mean (±SD).

**Figure** **3**a,b shows calculated concentrations with respect to time that followed the same trend as in Figure 2. Examination of caffeine concentrations for actual tests on the fetal side (Figure 3a) reveals a more conclusive result than that for concentrations represented by areas under the curve (Figure 2a). In this study, we investigated both the steady‐state concentration and with the amount of time required to reach this condition. Knowing steady‐state concentrations on the maternal and fetal sides will assist in verifying the safest dose of caffeine to be taken by a mother when a certain concentration is described in terms of the safe concentration level in the fetus. Since this system was used only as a proof‐of‐concept to verify the caffeine transport across the placenta in vitro, only one caffeine concentration (0.25 mg mL^−1^) within the safe amount of caffeine according to FDA was tested. After 5 h, the caffeine concentration began to reach a steady‐state of 0.0032 mg mL^−1^, and between 5 and 7.5 h, it maintained an average of 0.0033 mg mL^−1^ in steady‐state. Fetal caffeine concentration in controlled tests reached its peak at *t* = 6.5 h, and between 6.5 and 7.5 h it achieved its steady‐state at an average of 0.0179 mg mL^−1^. Similarly, analyzing the caffeine on the maternal side (Figure 3b) shows that steady‐state for the actual tests was achieved between 6 and 7.5 h at a value of 0.1513 mg mL^−1^ (average). During the controlled experiment, it was noted that steady‐state was achieved after 7 h at a caffeine concentration of 0.1307 mg mL^−1^. After 7.5 h, we observed cells detaching from the membrane. At this point, we concluded that the system could no longer provide a confluent layer of cells and would therefore not adequately represent a system to be used for actual experiments. Such failure could be attributed either to effects of caffeine on the cells or flow phenomenon inside the channels. Further studies are required to identify or confirm reasons for underlying cell detachment. Steady‐state was defined at the point in time or time range where caffeine concentrations seemed to maintain a steady value with respect to time while caffeine continued to be introduced into the maternal side at a constant flow rate.

A study on a physiologically based human model of a pregnant woman concluded that, after introducing caffeine, the concentration increased until it reached a steady‐state value.^33^ In that study, multiple doses were introduced, and each time the peak concentration was increased until it reached a steady‐state condition. We introduced about 0.0938 mg of caffeine to the maternal side within 7.5 h through medium perfusion, and while we can relate our tests to a similar study using multiple doses over time, the doses were continuously given, possibly explaining why Figure 2a,b has multiple peaks but only reached a single steady‐state condition. Since we continuously perfused caffeine diluted medium for 7.5 h, continuous perfusion resulted in multiple peaks with only a single steady‐state region. In addition, concentrations reported in controlled experiments were significant in the absence of caffeine on either the maternal side or the fetal side because caffeine that perfused to the maternal side should come from either the maternal side or the fetal side; we believe this is due to caffeine absorption to the edges on poly(dimethylsiloxane) (PDMS) side walls in the maternal channel. This was not significantly observed in actual experiments due to the cell coverage in channels.

While the collected volume might have only a minimal error, even this small error could affect the final calculated value. For example, while the expected volume within a 30 min perfusion period is 25 µL (with a flow rate of 50 µL h^−1^), only a small error in volume could dilute the medium with an incorrect volume of methanol. This error was minimized by measuring the volume of the sample collected during each 30 min period diluted with the correct amount of methanol. In Figure 2a, the number of counts measured at later time points (i.e., *t* = 6 h) at the fetal side showed the same standard deviation order value as the average. This was observed at early time points (i.e., *t* = 2 h) on the maternal side, as shown in Figure 2b. Fluctuations of caffeine concentrations on the both maternal and fetal sides at later time points could be attributed to cell detachment, but earlier fluctuation of the maternal concentration could be a result of different medium dilutions with methanol, as mentioned earlier. Further studies are needed to find specific reasons for these errors. In Figure 3b, it was noted that the highest calculated caffeine concentration (0.2591 mg mL^−1^) was detected at *t* = 1.5 h. While this value is greater than the caffeine concentration introduced to the maternal side (0.25 mg mL^−1^), during the calculation process the fitted curves (in Figure 3c,d) were made with a 95% confidence level and that error could affect the caffeine concentrations calculated on both maternal and fetal sides.

#### 2.2.2 Rate Transfer of Caffeine

**Figure 4:**
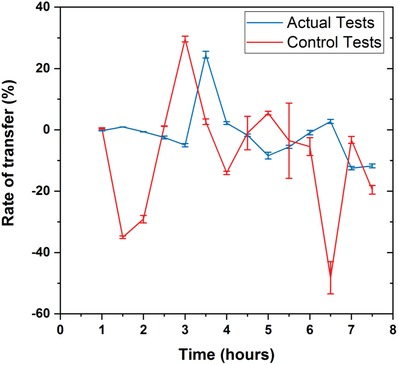
The rate of caffeine transfer calculated at every 30 min for both the actual (with cells) and control (without cells) tests. The rates were calculated cumulatively using the values at previous time point as the initial‐maternal and initial‐fetal concentrations (i.e., if the rate of transfer is calculated at *t* = 6 h, values at *t* = 5.5 h were used as the initial‐maternal and initial‐fetal concentrations). *n* = 3 independent experiments. Data were presented as mean (±SD).

The rate of caffeine transfer was calculated for both maternal and fetal sides using the following equation (Equation (3)):

(3) $$\%RT\;=\;\frac{\Delta C_{f}}{\Delta C_{m}}\;\times \;100$$

where Δ*C*_f_ and Δ*C*_m_ represent the change in caffeine concentrations in the fetal and maternal channels, respectively, during perfusion. Initial and final caffeine concentrations from both the maternal and fetal sides were used when calculating the values for Δ*C*_f_ and Δ*C*_m_. To calculate the initial maternal and fetal caffeine concentrations, the values at a previous time point were used for both the actual and controlled experiments (**Figure** **4**). Calculated rates were used to measure the change in rate of caffeine transfer with respect to the rate of caffeine transfer calculated at the previous time point (i.e., if the rate of caffeine transfer was calculated at *t* = 5 h, the values at *t* = 4.5 h were used as the initial concentrations).

As shown in Figure 4, the transfer rates for actual tests (with cells) reflected less fluctuation when compared to the rate calculated for controlled tests (without cells), when more caffeine was introduced into the system. In actual experiments, the transfer rates calculated from *t* = 1 h to *t* = 3 h show a gradual decrease, followed by a sudden increase and another gradual decrease in caffeine transfer rate observed between *t* = 3 h and *t* = 5 h. Similar patterns were seen in transfer rates for the controlled tests from *t* = 1.5 h to *t* = 4 h. The frequent fluctuations were attributed to the constant perfusion of caffeine at 50 µL h^−1^ to the maternal side, although further investigation is needed to find the exact reasons for such fluctuations. It has been previously reported that the transfer rate of caffeine across a placental barrier depends also on its physiochemical properties such as its size (molecular weight), ionization yield, lipophilicity (Log P), and protein binding.^34^ High‐permeability coefficients are observed for small polar molecules because such compounds pass readily through lipid membranes.^35^

Assertions about the amount of caffeine safe for consumption during pregnancy vary depending on the study referenced, and the FDA, taking into account for all the variations for this value has stated that any amount less than 300 mg per day is safe for pregnant women.^29^ In our study, we used a concentration of 0.25 mg mL^−1^ of caffeine, less than the FDA‐specified amount (300 mg per day = 0.67 mg mL^−1^), for perfusion analysis.

## 3 Conclusions

In this study, we successfully fabricated a placenta‐on‐a‐chip device using PDMS soft lithography techniques. After confirming that we had a confluent layer of cells, we used it to conduct caffeine transport analysis. A caffeine calibration curve was initially established to quantify the caffeine in collected media from both maternal and fetal channels. Using an integrated equation, caffeine concentrations in each media were calculated for each sample over a 7.5 h time span, producing a result showing that caffeine concentration on the fetal side increases until it reaches a steady‐state condition. In actual tests (with cells), caffeine concentration on the fetal side reached a steady‐state of 0.0033 mg mL^−1^, while in controlled tests it reached the steady‐state of 0.0179 mg mL^−1^ in the interval between 6.5 and 7.5 h. On the maternal side, while initial concentrations fluctuated, they reached steady‐state within 7.5 h. The steady‐state value was 0.1513 mg mL^−1^ between 6.5 and 7.5 h for the actual tests and 0.1307 mg mL^−1^ after 7 h for the controlled tests. This result clearly warrants further investigation on perfusing different caffeine concentrations to the maternal interface and the way they affect transfer rates.

## 4 Experimental Section

*Cell Culture*: HUVECs (Lonza) were chosen to represent the cells at the fetal interface. The cells were cultured with endothelial basal medium (EBM, R&D Systems), supplemented with Endothelial cell growth supplement (R&D Systems) containing fetal bovine serum (FBS). BeWo (ATCC) was selected from a variety of trophoblast cell lines based on its adhesive properties, functionality, and phenotype.^36^ BeWo was used to represent the cells at the maternal interface. The cells were cultured in Kaighn's Modification of Ham's F‐12K medium (Thermofisher), supplemented with 10% FBS (Thermofisher). Both cell lines were maintained in an incubator at 37 °C with 5% CO_2_ in air until they were 80–90% confluent.

**Figure 5:**
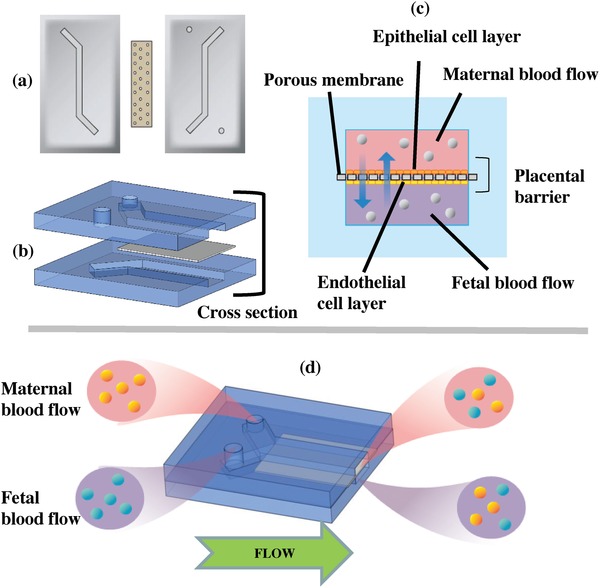
The placenta‐on‐a‐chip consists of two layers of PDMS separated with a porous membrane and channel on each side. a) Top and bottom layers with a porous membrane separating the channels before being attached. b) Channels aligned and ready to be attached and the porous membrane placed in between the layers to separate the midsections of each channel (where two cell layers interact). c) Cross‐sectional view of the channels. d) Experimental concept that shows the maternal and fetal bloodstreams perfused through the channels (not to scale).

*Design and Fabrication of the Chip*: The placenta‐on‐a‐chip device (**Figure** **5**) consisted of two microchannels (height: 100 microns; width: 400 microns) fabricated on two PDMS layers. An SU‐8 mold for the chip was created using standard soft lithography techniques. The silicon wafer mold was placed in a 15 cm diameter petri dish, and then a 10:1 w/w mixture of PDMS base and curing agent solution (Dow Corning) were introduced into the mold.^37^, ^38^ Once the PDMS had solidified at room temperature, it was cut and peeled away from the mold to separate it into upper and lower layers. To provide fluid access for each individual channel, inlet/outlet holes (1 mm diameter) were created using a biopsy punch. A 0.4 micron pore‐sized polyester track etched (PETE) membrane from the membrane inserts (Corning) was used to represent the barrier between fetal and maternal bloodstreams. The membrane covered the mid‐section of the lower channel before both layers were treated with plasma for 1 min, and the two PDMS layers were then aligned, attached, and left overnight to perfectly cure the bond. 1/16 ft diameter PEEK tubes (IDEX Health and Science) were then inserted into the inlets and outlets, attached to 0.062 × 0.125 in laboratory tubing (DOW Corning), then left overnight before use. After the layers were permanently bonded, the chip was UV‐sterilized for 20 min. Entactin–collagen IV–laminin (E–C–L, Millipore) solution was prepared from a diluted solution of E–C–L with a sterile serum‐free medium for each cell line up to a final concentration of 10 µg mL^−1^. Both sides of the membrane were initially coated with E–C–L solution, after which the chips were refrigerated overnight at 4 °C. Prior to cell seeding, the channels were washed twice with phosphate‐buffered saline (PBS) to remove excess E–C–L.

*Microfluidic Cell Seeding and Culturing on the Chip*: Once the HUVECs and BeWo cells reached 80–90% confluence, the cells were prepared for infusion. The density of the dissociated cells was adjusted to 5 × 10^6^ cells mL^−1^. The HUVECs were suspended in EGM medium, seeded into the lower channel, and incubated in an inverted position at 37 °C with 5% CO_2_ in air for 1 h to ensure reliable attachment to the membrane. Similarly, the BeWo cells suspended in F‐12K medium were introduced into the upper channel and incubated at the original position under similar conditions for 1 h. Once cell attachment was confirmed, the inlet of each channel was connected to 3 mL syringes (Becton, Dickinson and Company) filled with the respective growth media for each cell type seeded into the channels, after which the syringes were connected to a syringe pump driven at a constant volumetric flow rate of 50 µL h^−1^.

*Observing Live Cells*: The HUVECs and BeWo cells were stained with CellTracker green and CellTracker orange fluorescent probes (Life Technologies), respectively. Dissociated cells were incubated with staining diluted serum‐free medium (final working concentration of 0.5–25 × 10^−6^ m) at 37 °C with 5% CO_2_ for 45 min.

*Investigating the Barrier Permeability*: Three thousand megawatt fluorescein–dextran anionic probes (Invitrogen, ThermoFisher) were used to measure the barrier permeability function based on its transport between maternal and fetal channels. Fluorescein–dextran was first diluted in PBS to 100 mg mL^−1^ then brought to a final concentration of 0.1 mg mL^−1^ in F‐12K medium. F‐12K supplement for the maternal channel was replaced with dextran‐mixed F‐12K and perfused for 4 h. Flow from both maternal and fetal channels was collected each hour and the fluorescence intensity of the collected samples was analyzed using a microplate reader (BioTek Synergy 2).

*Cell Characterization for Analyzing Intercellular Junctions*: After confirming proliferation of cells on membranes inside the channels for a minimum of 3 days, the channels were rinsed twice with 0.1 m PO_4_ buffer, after which the cells were fixed in 4% paraformaldehyde and incubated at room temperature for 20 min. The channels were then washed thrice with PBS at 7 min increments. The channels were subsequently incubated at room temperature in a blocking solution created using 5% normal donkey serum as the normal blocking serum (NBS, Jackson Immuno Research Labs), 0.4% bovine serum albumin (BSA), and 0.2% Triton X‐100 for 60 min. Following incubation, primary antibodies (E‐cadherin and VE‐cadherin [Cell Signaling Technologies] for BeWo and HUVECs, respectively) were diluted in previously prepared blocking serum and incubated in each channel overnight at 4 °C. After being washed in PBS 4 times, the channels were incubated for 90 min with secondary antibodies and DAPI solution diluted in the same blocking solution. The channels were then rinsed with PBS 4 times with 8 min intervals between each rinse. After carefully separating the membrane from the chip, it was mounted to a coverslip and imaged with an inverted microscope (Zeiss Axio Observer Z1).

*Analysis of Caffeine Transport*: An LC/MS analytical method was used to determine the caffeine concentrations, using an Agilent Technologies 1100 Series advanced high‐performance liquid chromatography (HPLC) tandem mass spectrometer equipped with a Poroshell 120 EC‐C18 2.7 µm 4.6 mm × 50 mm (Agilent) column (W.M. Keck Metabolomics Laboratory, Iowa State University), to detect caffeine levels.^39^ This instrument is composed of a UV–vis capable diode array detector and an Agilent Technologies Mass Selective Trap SL detector equipped with an electrospray ion source.

To prepare the collected samples for runs, each sample was diluted at a ratio of 1:3 in methanol and vortexed for several seconds, after which the samples were centrifuged at 16 × *g* for 5 min and 100 µL from each sample was transferred to separate vials. The mobile phase was a mixture of water (80% with 0.1% acetic acid) and acetonitrile (20% with 0.1% acetic acid). The mass analyzer operated with an ESI source in positive ion mode, and the flow rate and injection volume were 0.75 mL min^−1^ and 5 µL, respectively. The quantification for caffeine was determined by measuring the intensity of protonated molecular ions of caffeine at *m/z* 195.

*Caffeine Transport across the Placental Barrier*: Calibration curves were initially developed so that the correlation could be used to calculate the amount of caffeine transported from the maternal side to the fetal side. Different caffeine (Sigma Aldrich) concentrations ranging from 0.00001 to 0.25 mg mL^−1^ in EGM and from 0.05 to 0.3 mg mL^−1^ in F‐12K were used to create calibration curves using the data collected via the LCMS method. Assuming that the area under the curve for the caffeine spike from chromatogram (example chromatogram as shown in Figures S1 and S2 in the Supporting Information) was proportional to the concentration of the caffeine in each medium, the two different graphs shown in Figure 3c,d were used to represent the correlation between concentration of caffeine and area under the curve. A 0.25 mg mL^−1^ caffeine solution in F‐12K medium was then introduced into the maternal side. Following a 1 h perfusion period, samples were collected after every 30 min from both the maternal and fetal outlets, and each sample was analyzed using the LCMS method to identify the exact amount of caffeine transported across the placental barrier.

*Quantification of Caffeine Concentrations and Transfer Rates*: Using Figure 3c,d, the caffeine concentrations for both the maternal and fetal sides were quantified and used to study the percentage increase of caffeine concentration in the fetal compartment over a period of medium perfusion. Equation (3) was used to calculate the rate of caffeine transfer.

## Conflict of Interest

The authors declare no conflict of interest.

## Supporting information

Supplementary
