# Supplementary material for: Placenta‐on‐a‐Chip: In Vitro Study of Caffeine Transport across Placental Barrier Using Liquid Chromatography Mass Spectrometry
Source: Glob Chall. 2019 Feb 18;3(3):1800112. doi: 10.1002/gch2.201800112 (PMC6436596; doi:10.1002/gch2.201800112)
Supplement: Supplementary file 1 — Supplementary [file GCH2-3-1800112-s001.pdf]

Copyright WILEY-VCH Verlag GmbH & Co. KGaA, 69469 Weinheim, Germany, 2019.

Global Challenges
Open Access

Supporting Information

for *Global Challenges,* DOI: 10.1002/gch2.201800112

Placenta-on-a-Chip: In Vitro Study of Caffeine Transport across Placental Barrier Using Liquid Chromatography Mass Spectrometry

*Rajeendra L. Pemathilaka, Jeremy D. Caplin, Saurabh S. Aykar, Reza Montazami, and Nicole N. Hashemi**

Copyright WILEY-VCH Verlag GmbH & Co. KGaA, 69469 Weinheim, Germany, 2019.

Supporting Information

Placenta-on-a-Chip: in vitro Study of Caffeine Transport across Placental Barrier Using Liquid Chromatography Mass Spectrometry

Rajeendra Pemathilaka [1], Jeremy Caplin [1,2], Saurabh Aykar [1], Reza Montazami [1], and Nicole N Hashemi [*,1,3]

[1] Department of Mechanical Engineering, Iowa State University, Ames, IA 50011, USA

[2] Petit Institute for Bioengineering and Bioscience, Georgia Institute of Technology, Atlanta, GA 30332, USA

[3] Department of Biomedical Sciences, Iowa State University, Ames, IA 50011, USA

[*] E-mail: nastaran@iastate.edu

R. L. Pemathilaka, J. D. Caplin, S. S. Aykar, Prof. R. Montazami, Prof. N. N. Hashemi

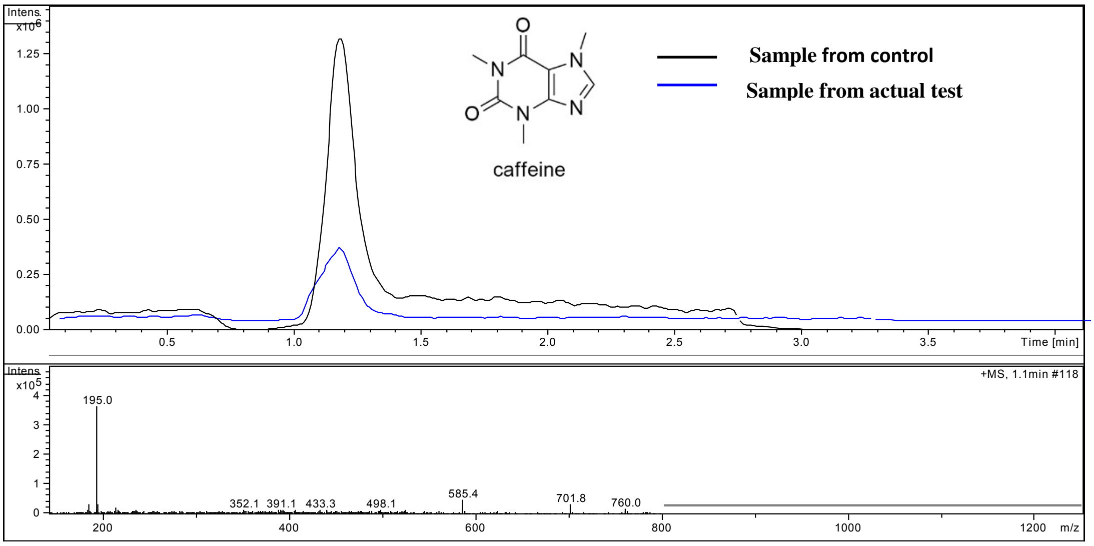

Figure S1: Chromatogram of caffeine for samples collected from the fetal compartment at t = 7.5 hours. Actual tests were conducted on cell-seeded chips with perfusing EGM and F-12K, while controlled tests were conducted on chips with a bare membrane and perfusing EGM and F-12K.

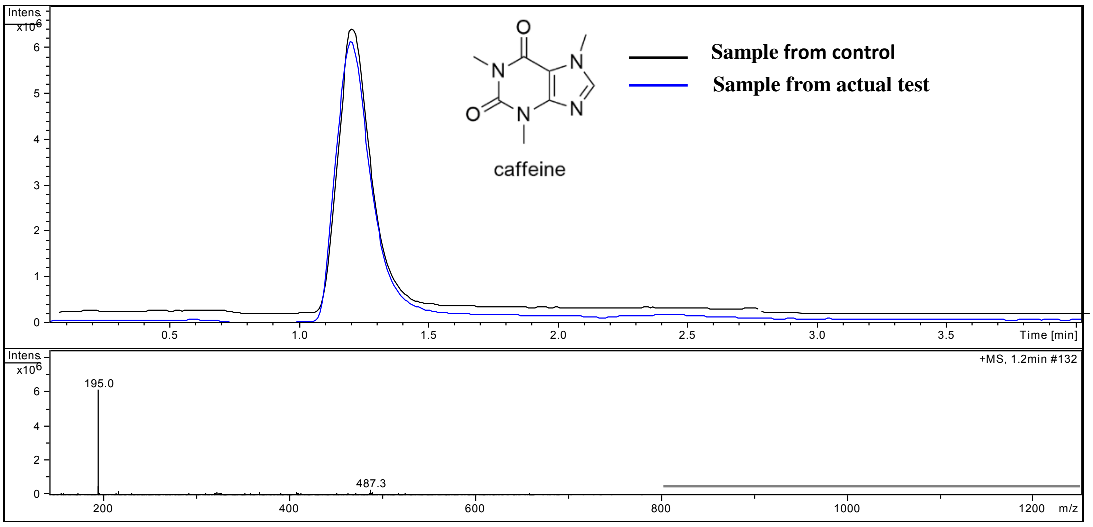

Figure S2: Chromatogram of caffeine for samples collected from the maternal compartment at t = 7.5 hours. Actual tests were conducted on cell-seeded chips with perfusing EGM and F-12K, while controlled tests were conducted on chips with a bare membrane and perfusing EGM and F-12K.
